# Editorial: Advances towards precision medicine in pediatric-onset inflammatory bowel disease

**DOI:** 10.3389/fmed.2025.1765757

**Published:** 2026-01-13

**Authors:** Jan De Laffolie, Almuthe Christina Hauer

**Affiliations:** 1 Department of General Pediatrics and Neonatology, University of Giessen, Giessen, Germany; 2 Department of Pediatrics and Adolescent Medicine, Medical University of Graz, Graz, Austria

**Keywords:** microbiome-based interventions, patient-reported outcomes, pediatric-onset IBD, precision medicine, therapeutic drug monitoring, treat-to-target strategies

## Key points

Pediatric-onset IBD is increasingly common and often presents with more severe, extensive disease than in adults.

- Precision medicine aims to deliver the right treatment, at the right time, to the right child, based on individual clinical, laboratory, imaging, and microbiome data.
- Current studies highlight progress in risk prediction, treat-to-target strategies, therapeutic drug monitoring, microbiome-based interventions, and patient-reported outcomes.
- Significant gaps remain, including limited sample sizes, single-center cohorts, preclinical evidence, and equity of access to advanced therapies.
- Achieving truly individualized, evidence-based, patient-centered care requires collaboration, high-quality data, and thoughtful implementation in all healthcare settings.

Pediatric-onset Inflammatory Bowel Disease (pIBD) has shifted from being a rare curiosity to a central challenge in modern Pediatric Gastroenterology. Its incidence and prevalence are rising worldwide (1), and children frequently present with more extensive and more aggressive disease than adults. The consequences are not confined to the gut: growth failure, delayed puberty, disturbed schooling, family strain, and long-term psychosocial sequelae are all part of the lived reality of many patients, families and carers (2, 3). Against this background, the aspiration of “precision medicine”—to deliver the right treatment, at the right time, to the right child, with a clearly defined therapeutic target—is not a fashionable slogan but a clinical necessity (4).

The Frontiers Research Topic on “*Advances Towards Precision Medicine in Pediatric-onset Inflammatory Bowel Disease*” brings together seven contributions that address different facets of this agenda. They range from predictive nomograms and therapeutic drug monitoring to patient-reported outcomes, experiences with small molecules, microbiome-based concepts and a case report illustrating diagnostic complexity. Taken together, they offer a snapshot of where the field currently stands: moving in the direction of individualized patient- centered care, yet still with some way to go toward data-driven, precision-medicine learning health systems in pIBD.

A first cluster of papers is centered on treat-to-target concepts and risk prediction in Crohn's disease. One study develops a nomogram to predict small-bowel mucosal healing in children by combining clinical and laboratory variables. The underlying clinical question is highly pragmatic: can we estimate the probability of mucosal healing in the small intestine without subjecting every child to repeated capsule procedures, and can we do so in a way that meaningfully supports decisions on therapy intensification or de-escalation? By transforming several routinely available parameters into an individualized risk estimate, the nomogram provides a structured tool to support decisions.

A second Crohn's Disease-focused contribution looks at a particularly challenging phenotype: perianal fistulising disease. The authors analyse long-term infliximab therapy in children and adolescents with perianal fistulas, systematically measuring trough levels and correlating them with radiological healing on pelvic MRI. The key message is intuitively convincing and clinically important: higher infliximab trough concentrations, both during induction and maintenance, are associated with radiological fistula healing—a stricter and more meaningful endpoint than clinical closure alone. For everyday practice, this supports the widely held view that “standard” anti-TNF dosing may be insufficient in complex perianal disease and that proactive therapeutic drug monitoring with higher target levels is increasingly recognized.

The Research Topic also broadens the therapeutic and conceptual toolkit for ulcerative colitis (UC). One clinical series explores the off-label use of tofacitinib, a Janus kinase inhibitor, in pediatric UC patients with concomitant arthropathy. The authors show that tofacitinib can achieve meaningful improvement in both intestinal and articular disease in selected children who have often exhausted other therapeutic options and require off-label treatment.

Another article is devoted to a rapidly evolving area of interest: the role of the gut microbiome, and specifically *Akkermansia muciniphila*, in UC. The mini-review summarizes mechanistic data suggesting that *A. muciniphila* can strengthen the mucus layer, modulate tight junctions and influence mucosal immune responses. Experimental colitis models demonstrate that certain *Akkermansia* preparations can ameliorate inflammation, improve barrier function and alter cytokine profiles in a favorable direction. In the pediatric UC context, such strain-level microbiome manipulation offers an appealing future avenue for precision therapy: yet, as the review rightly acknowledges, we are not there yet. Pediatric interventional trials are lacking; long-term safety remains unknown and much of the evidence is still preclinical. The translational gap between mechanistic plausibility and routine clinical use is therefore considerable.

The question of infection risk stratification is addressed in a study that constructs a nomogram for predicting *Clostridioides difficile* infection (CDI) in children with UC. CDI is a well-recognized complication in pediatric IBD, associated with more severe flares, prolonged hospitalizations and a higher need for surgery. However, not all children carry the same risk. By analyzing a UC cohort and identifying clinical and laboratory predictors of CDI, the authors develop a simple graphical risk score that can be used at the bedside to estimate an individual child's probability of developing CDI. This potentially allows clinicians to prioritize early testing, isolation procedures and antimicrobial stewardship measures in those most at risk, rather than applying a uniform, often inefficient approach. As with any prediction tool, internal validation is encouraging but insufficient. External validation in independent, multinational cohorts, as well as implementation studies that test whether using the nomogram improves outcomes or resource allocation, will be crucial next steps.

Finally, the case report of an intra-abdominal mass in a young patient with Crohn's disease serves as a reminder that precision medicine begins with precise clinical reasoning and appropriate diagnostic procedures. The narrative illustrates how atypical presentations in known IBD patients require an open, multidisciplinary mind-set.

What, then, are the common strengths of this Research Topic? First, all contributions are closely aligned with clinically relevant questions that arise daily in a pIBD clinic: how high infliximab levels need to be to really heal fistulas; whether we can safely avoid repeating capsule endoscopy in a child; whether we may rely on PROMIS scores to monitor how a teenager is coping with their Crohn's disease; which UC patients merit particular vigilance for C. difficile; and when is a JAK inhibitor a reasonable escalation step in a child with refractory UC and disabling arthritis?

Second, the methodological approaches, while mostly conventional, move the field beyond descriptive epidemiology—by building nomograms, quantifying PRO responsiveness, linking drug exposure to objective healing, and translating mechanistic microbiome insights into therapeutic concepts.

The limitations of the Research Topic are equally instructive. Many of the cohorts are single-center, reflecting the reality that pIBD remains relatively rare and that tertiary institutions often act as the main hubs of expertise and research. Sample sizes are correspondingly modest, reducing statistical power, limiting subgroup analyses and increasing vulnerability to confounders and indication bias. Observational designs predominate, especially in the therapeutic and TDM studies, so causal inference is necessarily cautious. The prediction models for mucosal healing and CDI are internally validated but not yet externally tested, and they treat risk as a static snapshot rather than a dynamic quantity that fluctuates with treatment changes and disease course over time.

Finally, questions of equity and generalisability remain largely unanswered. Most data derive from high-income settings with ready access to biologics, advanced imaging, therapeutic drug monitoring and endoscopic infrastructure. The reality of caring for a child with IBD in regions with limited resources, fragmented health systems or substantial financial barriers looks very different, and precision medicine must not become synonymous with care for the privileged few.

Despite these caveats, this Research Topic represents a meaningful step forward. It shows that in several key areas pediatric IBD is already moving beyond “one-size-fits-all” algorithms. The contributions provide clinicians with new lenses through which to view familiar problems and offer methodologic starting points for more ambitious, integrated projects.

In that sense, the articles gathered here are best seen not as the culmination of the precision-medicine journey in pediatric IBD, but as waypoints. They give us direction, illustrate what is possible and make visible what is missing. For clinicians, researchers and families striving for truly individualized, evidence-based patient-centered care, they offer not only encouragement but also a compelling mandate: to accelerate collaboration, expand high-quality data generation and ensure that innovations reach every child who needs them. Precision medicine in pIBD is no longer an aspiration on the horizon—it is the path ahead, and the work begins now.
